# Averting flood-related deaths and injuries from hurricanes: enhancing hospital resilience

**DOI:** 10.1016/j.lana.2024.100930

**Published:** 2024-10-23

**Authors:** Attila J. Hertelendy, Gregory R. Ciottone

**Affiliations:** a Department of Information Systems and Business Analytics, College of Business, Florida International University, Miami, FL, USA; b Disaster Medicine Fellowship, Department of Emergency Medicine, Beth Israel Deaconess Medical Center, Boston, MA, USA; c Harvard Medical School, Boston, MA, USA

As temperatures rise globally the frequency and severity of cyclones and associated flooding events are increasing and resulting in increased morbidity and mortality.^1^, ^2^, ^3^ Throughout the Americas hurricanes pose significant threats to public health and healthcare infrastructure, often overwhelming local response capacities.^4^ Hurricane Helene made landfall in northwest Florida as a category 4 storm on September 26, 2024, causing historic flooding across six states surpassing 230 deaths mostly due to flooding, making it one of the most devastating hurricanes to hit the US mainland since Hurricane Katrina in 2005.^5^ Subsequently, less than 2 weeks later Hurricane Milton made landfall along the western coast of Florida in Sarasota County on October 9th, 2024 as a Category 3 storm. In the midst of the hurricane an unprecedented 130 tornado warnings were issued with several tornadoes touching down resulting in four confirmed deaths.^6^

Applying best practices gleaned from recent hurricanes, healthcare leaders and policymakers can implement a comprehensive approach to strengthening healthcare system resilience against these severe storms and resultant floods. The following strategies are derived from both best practices and the authors’ first-hand experience aimed at reducing morbidity and mortality, with a particular emphasis on ensuring hospital continuity of operations during these catastrophic events.

## Community-level interventions

Effective flood impact mitigation involves key interventions that begin at the community level, including enhanced early warning systems, community-based alerts, and robust evacuation planning, particularly for vulnerable populations like the elderly and disabled. Considering recent experiences with Hurricane Helene, communities must also address the threat of disinformation campaigns that can undermine government response efforts.^7^

## Healthcare system preparedness

Hospitals must be prepared for long-term continuity of operations even if directly affected by the disaster. The literature has commented on the increasing frequency and duration of extreme weather events, and the need to prepare for multiple crises occurring simultaneously.^2^^,^^8^ Hurricanes Helene and Milton occurring less than 2 weeks apart in the same geographical area is an example of compounding crises which may overwhelm healthcare systems, as might multiple crises occurring simultaneously, such as a cyberattack during a natural disaster.

## Resilient hospital infrastructure

Hospitals in hurricane-prone areas should be made flood resilient, including elevating critical infrastructure and implementing redundant systems. Essential components such as electrical equipment, backup generators, and medical gas supplies should be located above potential flood levels. Multiple backup power sources, water supplies, and communication systems should be installed to ensure continuity of care. During Hurricane Harvey, Memorial Hermann Hospital in Houston maintained operations despite severe flooding, largely due to its elevated critical infrastructure.^9^ In response to both Hurricane Helene, and Hurricane Milton, Tampa General Hospital, the regions only level 1 trauma hospital successfully deployed their “Aquafence” barrier, which prevented the storm surge from flooding the hospital, allowing the facility to continue normal operations.^10^

## Comprehensive emergency response plans

Hospitals must develop detailed emergency response plans that encompass three critical areas: staff management, patient evacuation protocols, and supply chain management. Clear evacuation protocols are also essential.^11^ The successful evacuation of 243 patients from NYU Langone Health during Hurricane Sandy offers valuable insight into executing large-scale hospital evacuations, underscoring the importance of well-planned and rehearsed evacuation procedures.^12^ Hurricane Helene's impact on supply chains reinforces the need for robust plans to maintain critical supplies and strategies for alternative delivery methods. The importance of this was exemplified by the shortage of IV fluids following the hurricane.

## Enhanced communication systems

Hospitals must implement robust and resilient communication infrastructure.^8^^,^^11^ This should include redundant technologies and emergency notification systems to ensure uninterrupted communications. Equally important is the implementation of interoperable systems that are compatible with local emergency services, facilitating coordinated responses and maintaining critical lines of communication.^2^

## Specialized flood response training

To enhance readiness, hospitals should implement comprehensive training programs for their staff that focus on flood-specific scenarios and include education on casualty care and infection control, such as managing waterborne diseases in flood-affected environments. Simulations may help staff practice these skills, improving their ability to respond effectively when actual floods occur.^13^

## Community health outreach

During floods, hospitals may need to extend their care beyond their physical boundaries. Developing robust community outreach strategies is crucial for providing essential healthcare services to flood-affected populations unable to reach the hospital. Two key components of effective outreach are mobile medical units and telemedicine capabilities.

Deployable mobile medical units bring vital healthcare services to victims cut-off by floodwaters. These units can provide emergency care, dispense medications, and offer basic medical treatments.^14^ Additionally, robust telemedicine systems can ensure continuity of care for patients while minimizing their need to travel through hazardous conditions. Telemedicine can facilitate remote consultations, monitoring of chronic conditions, and mental health support.

## Innovative approaches to flood resilience in healthcare

Artificial Intelligence (AI) is emerging as a powerful tool for flood-related disasters. AI-driven models can significantly improve pre-flood impact mapping, providing hospitals with crucial lead time to implement emergency protocols. Moreover, AI can play a vital role in optimizing hospital resource allocation during emergencies by processing real-time data on patient influx, supply levels, and staff availability. For instance, AI could help determine the most efficient evacuation routes or suggest the optimal distribution of medical resources across a flood-affected region.^15^

Unmanned Aerial Vehicles (UAVs), commonly known as drones, represent another innovative solution. Drones could deliver essential medical supplies to hospitals in need, including a variety of critical items like blood products or medical devices, ensuring that flood-affected hospitals continue providing necessary care. Furthermore, drones equipped with cameras and sensors could be used for rapid damage assessment of hospital infrastructure or to scout safe evacuation routes, providing valuable real-time information to hospital management and emergency responders.

As global warming continues to fuel the frequency and severity of hurricanes, the need for improved preparedness measures becomes increasingly urgent. Healthcare leaders and policymakers must collaborate to implement these strategies, ensuring that our communities and healthcare systems are ready to face the significant challenges of hurricane events.

## Contributors

AJH. was involved with conceptualization, project administration, original draft, review and editing.

GC contributed to the writing, original draft, review and editing.

## Declaration of interests

We declare no competing interests.
